# Supplementary material for: Estimating the functional dimensionality of neural representations
Source: Neuroimage. 2018 Oct 1;179:51–62. doi: 10.1016/j.neuroimage.2018.06.015 (PMC6057285; doi:10.1016/j.neuroimage.2018.06.015)
Supplement: functionaldimensionality_sm [file mmc1.pdf]

Supplemental Material: Estimating the functional dimensionality of neural representations

Christiane Ahlheim[a,*], Bradley C. Love[a,b,]

[a]Department of Experimental Psychology, University College London, 26 Bedford Way, London WC1H 0AP, United Kingdom
[b]The Alan Turing Institute, United Kingdom

1. Assessing effect of prior assumptions on model results

In addition to the simulations reported in the main paper, we here illustrate the effects that different prior assumptions regarding the functional dimensionality of a region have, depending on the true dimensionality and the noise level.

1.1. Methods

The simulated data reported here were created in the same way as described in the main paper. We simulated data for three different ground-truth dimensionalities (4, 8, and 12) and 10 steps of exponentially increasing noise-level. We ran a total of 10 of these simulations for each combination of ground-truth dimensionality and noise-level.

We built three different hierarchical Bayesian models, each with a different prior distribution over the parameter of the population dimensionality estimate μ. Each prior distribution was parametrized as a beta-distribution for which we set the two shape parameters to 1) 1 and 99, expressing a prior favoring low dimensionality estimates, 2) 99 and 1, which favors high dimensionality estimates, and 3) 50 and 50, which favors medium dimensionalities.

As before, we combined all posterior estimates of the single simulated participants' dimensionalities (parameter μ_i) across all simulated voxels, separately for each hierarchical model. The width of the distributions of these posteriors reflects the uncertainty of the estimated population dimensionality, and the distributions' means reflect the estimated population dimensionality.

1.2. Results and Discussion

Across 10 simulations of data with a ground-truth dimensionality of 4, 8, or 12 and ten different noise levels, we assessed the effect of three different priors on estimated dimensionalities.

We can see that the general pattern of results mirrors the results we found when modeling the data with a uniform prior (see Figure S1, cf Figure 5). For a low noise level, the estimated dimensionalities largely overlap with the ground-truth. With increasing noise, estimated dimensionalities deviate more strongly from the underlying ground-truth and shift towards a medium dimensionality. Furthermore, the uncertainty in the dimensionality estimates increases, reflected in the width of the

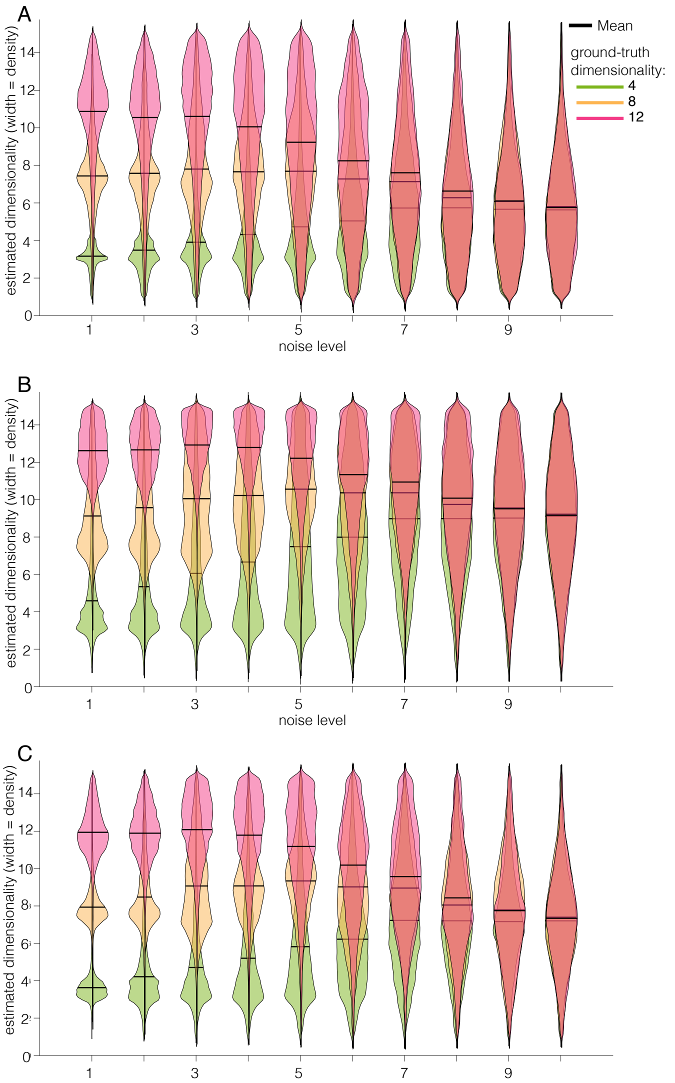

Figure 1: Effect of different prior distributions on estimated dimensionalities. A: Given a prior of $Beta(1, 99)$, that is, favoring low dimensionalities, estimated dimensionalities for high ground-truth dimensionalities tend to be under-estimated. With increasing noise, estimates become more uncertain and settle at an estimated dimensionality of approximately 6. B: Given a prior of $Beta(99, 1)$, that is, favoring high dimensionalities, estimated dimensionalities for low ground-truth dimensionalities tend to be over-estimated. With increasing noise, estimates become more uncertain and settle at an estimated dimensionality of around 9. C: Given a prior of $Beta(50, 50)$, that is, favoring medium dimensionalities, estimated dimensionalities match closely the ground-truth dimensionalities for low noise and become more uncertain as the noise increases, settling at a value of approximately 8.

*Corresponding author

distributions. If we compare the results from the three different models implementing different priors, we see that estimated dimensionalities given a prior favoring high values tend to be higher, with a larger uncertainty for ground-truth low dimensionalities, whereas estimated dimensionalities given a prior favoring low values show the opposite pattern. However, all posterior distributions always include the ground-truth dimensionality, which highlights the robustness of our method given reasonable prior assumptions.

2. Identifying areas carrying functional dimensionality

With the first dataset from a category learning study by Mack et al. (2013), we aimed to identify areas carrying functional dimensionality and compare them with the areas found by the original authors' model-based analysis.

2.1. Methods

Pre-processing of the data was carried out using SPM12 (Penny et al., 2006). Functional EPI data were motion-corrected with respect to the mean-image, T1 weighted anatomical scans were realigned to the EPI images, and both functional and anatomical images were normalized to MNI space with a voxel-resolution of $3 \times 3 \times 3$. Data were high-pass filtered at 128Hz to account for slow signal drifts. Beta estimates were derived from a GLM containing one regressor per stimulus (16 regressors in total), convolved with the HRF. Motion regressors were included in the GLM as covariates of no interest. Temporal autocorrelations were accounted for by implementing an autoregressive model (AR-1) during parameter estimation. Residuals of the GLM for each timestep were saved and used later on for pre-whitening of the data.

3. Using functional dimensionality to assess sensitivity to stimulus features

Using data from a study of real-world categories using photographic stimuli by Bracci and Op de Beeck (2016), we tested whether different regions show functional dimensionality in response to different stimulus features, depending on how the stimulus-space is summarized.

3.1. Methods

During the experiment of the second dataset, participants performed a 1-back real-world size judgment task. Each participant completed two sessions (on two different days) of eight runs. For one participant, four runs were lost. Each image was presented twice per run.

Pre-processing of the data was carried out using SPM12. Functional EPI data were motion-corrected with respect to the mean-image, T1 weighted anatomical scans were realigned to EPI images, and both functional and anatomical images were normalized to MNI space with a voxel-resolution of $3 \times 3 \times 3$. Data were high-pass filtered at 128Hz to account for slow signal drifts. We aimed to test if our method could be applied to assessing qualitative coding differences across the brain by varying how the stimulus space is summarized. In line with the authors original analysis, we tested for differences depending on whether the stimuli were averaged to emphasize their category or shape information. To that end, we constructed two separate GLMs. The first GLM (catGLM) was composed of one regressor per category (six in total), thus averaging across objects shapes. The second GLM (shapeGLM) consisted of nine different regressors, one for each shape, averaging neural responses across object categories. In both GLMs, regressors were convolved with the HRF and six motion-regressors as covariates of no interest were included.

Dimensionality was estimated separately for both GLMs. We ran a wholebrain searchlight with a 7mm sphere on the beta estimates of the respective GLM, again pre-whitening and mean-centering voxel patterns within each searchlight before estimating the dimensionality. Reconstruction correlations were averaged across runs for each participant and tested for significance across participants using FSL's randomise function (Winkler et al., 2014). Results were FWE corrected using a TFCE threshold of $p < .05$.

4. Measuring task-dependent differences in dimensionality

In this third dataset, we considered whether the underlying dimensionality of neural representations changes as a function of task. In Mack et al. (2016), participants learned a categorization rule over a common stimulus set that either depended on one or two stimulus dimensions. We predicted that the estimated functional dimensionality, as measured by our hierarchical Bayesian method, should be higher for the more complex categorization problem, extending the original authors' findings.

4.1. Methods

Each participant completed twelve functional runs in total, of which four were on type I problem and four on type II problem (the first four runs served as familiarization with the stimuli).

Pre-processing of the data was carried out using SPM12 (Penny et al., 2006). Functional EPI data were motion-corrected with respect to the mean-image, T1 weighted anatomical scans were realigned to EPI images, and both functional and anatomical images were normalized to MNI space with a voxel-resolution of $3 \times 3 \times 3$. Data were high-pass filtered at 128Hz to account for slow signal drifts. Beta estimates were derived from a GLM containing one regressor per stimulus (8 regressors in total), convolved with the HRF. Six motion regressors were included in the GLM as covariates of no interest. Temporal autocorrelations were accounted for by implementing an autoregressive model during parameter estimation. Residuals of the GLM for each timestep were saved and used later on for pre-whitening of the data.

We defined a region of interest (ROI) in the left and right LOC based on voxels that showed increased activation with trial onset, based on a separate GLM with only a single regressor

modeling all trials ($p < .001$, uncorrected; left LOC: 120 voxels, right LOC: 220 voxels). Using an ROI instead of a searchlight approach allowed us to estimate the degree of functional dimensionality rather than only identifying which areas showed functional dimensionality. We estimated dimensionality across these two ROIs separately for the two different categorization tasks. To reduce the impact of category-learning on the estimated dimensionality, the first functional run of each problem type was excluded from the analysis, resulting in three runs for each problem.

Bracci, S. and Op de Beeck, H. (2016). Dissociations and associations between shape and category representations in the two visual pathways. *Journal of Neuroscience*, 36(2):432–444.

Mack, M. L., Love, B. C., and Preston, A. R. (2016). Dynamic updating of hippocampal object representations reflects new conceptual knowledge. *Proceedings of the National Academy of Sciences of the United States of America*, 113(46):13203–13208.

Mack, M. L., Preston, A. R., and Love, B. C. (2013). Decoding the brain's algorithm for categorization from its neural implementation. *Current Biology*, 23(20):2023–2027.

Penny, W., Friston, K., Ashburner, J., Kiebel, S., and Nichols, T. (2006). *Statistical Parametric Mapping: The Analysis of Functional Brain Images: The Analysis of Functional Brain Images*, volume 8. Academic press.

Winkler, A. M., Ridgway, G. R., Webster, M. A., Smith, S. M., and Nichols, T. E. (2014). Permutation inference for the general linear model. *NeuroImage*, 92:381–397.
